# Real-Time Reverse Transcription–Polymerase Chain Reaction Assay for SARS-associated Coronavirus

**DOI:** 10.3201/eid1002.030759

**Published:** 2004-02

**Authors:** Shannon L. Emery, Dean D. Erdman, Michael D. Bowen, Bruce R. Newton, Jonas M. Winchell, Richard F. Meyer, Suxiang Tong, Byron T. Cook, Brian P. Holloway, Karen A. McCaustland, Paul A. Rota, Bettina Bankamp, Luis E. Lowe, Tom G. Ksiazek, William J. Bellini, Larry J. Anderson

**Affiliations:** * Centers for Disease Control and Prevention, Atlanta, Georgia, USA

**Keywords:** SARS, Coronavirus, Real-Time PCR, RT-PCR

## Abstract

A real-time reverse transcription–polymerase chain reaction (RT-PCR) assay was developed to rapidly detect the severe acute respiratory syndrome–associated coronavirus (SARS-CoV). The assay, based on multiple primer and probe sets located in different regions of the SARS-CoV genome, could discriminate SARS-CoV from other human and animal coronaviruses with a potential detection limit of <10 genomic copies per reaction. The real-time RT-PCR assay was more sensitive than a conventional RT-PCR assay or culture isolation and proved suitable to detect SARS-CoV in clinical specimens. Application of this assay will aid in diagnosing SARS-CoV infection.

In late 2002, a life-threatening febrile respiratory illness appeared in Guangdong Province, China, and quickly spread throughout Asia and to other parts of the world (*1*–*4*). Designated “severe acute respiratory syndrome” (SARS), the etiologic agent was later identified as a hitherto unrecognized coronavirus (SARS-CoV) (*5*,*6*). A diagnosis of SARS is based primarily on clinical and epidemiologic criteria, but many respiratory viruses can cause similar symptoms, and therefore rapid, reliable diagnostic tests for SARS-CoV infection were needed. In response to this need, three types of diagnostic tests for SARS-CoV were quickly developed: tissue culture isolation, antibody detection, and reverse transcription-polymerase chain reaction (RT-PCR) assays.

A variety of RT-PCR assays were developed during the epidemic for SARS-CoV (*1*,*5*–*8*), including a commercial ready-to-use RT-PCR kit (Artus Biotech, Hamberg, Germany). Early assays based on conventional designs that required postamplification product processing (e.g., gel electrophoresis), were time-consuming and prone to false-positive results from amplicon contamination. Conversely, real-time RT-PCR assays based on detecting and quantifying a fluorescent signal generated during amplification do not require postamplification processing and therefore eliminate one potential avenue for template contamination.

A variant of the real-time format, based on TaqMan probe hydrolysis technology (Applied Biosystems, Foster City, CA), has been shown to provide sensitive, specific, and quantifiable results in viral diagnostic assays (*9*) and has been used successfully to study emerging virus infections (*10*,*11*), including SARS (*6*,*12*). In response to the SARS public health emergency, we developed and evaluated a TaqMan real-time RT-PCR assay based on three distinct targets in the SARS-CoV genome for rapid deployment to the National Laboratory Response Network for Bioterrorism (LRN) (http://www.cdc.gov/programs/bio.htm).

## Materials and Methods

### Clinical Specimens

A total of 177 clinical specimens collected from 66 patients who met the SARS case definition (*13*) were used in this study. Specimens included oro- and nasopharyngeal swabs (dry and in viral transport media), sputa, nasal aspirates and washes, bronchoalveolar lavage, and lung tissue specimens collected at autopsy. Specimen processing was performed in a class II biological safety cabinet using biosafety level three (BSL3) work practices. Three 100-μL aliquots of each specimen were distributed to vials each containing 900 μL of NucliSens lysis buffer (bioMérieux, Durham, NC) and stored at –70°C until testing.

### Virus Culture

Vero E6 cells were inoculated with clinical specimens and observed for cytopathic effect, consisting of cell rounding with a refractive appearance followed by detachment from the flask surface (*5*). Plaque titrations were conducted by standard methods (*14*).

### Nucleic Acid Extraction

Nucleic acids were recovered from clinical specimens using the automated NucliSens extraction system (bioMérieux). Following manufacturer’s instructions, specimens received in NucliSens lysis buffer were incubated at 37°C for 30 min with intermittent mixing, and 50 μL of silica suspension, provided in the extraction kit, was added and mixed. The contents of the tube were then transferred to a nucleic acid extraction cartridge and processed on an extractor workstation. Approximately 40–50 μL of total nucleic acid eluate was recovered into nuclease-free vials and either tested immediately or stored at –70°C.

### Primers and Probes

Multiple primer and probe sets were designed from the Urbani strain of SARS-CoV polymerase 1b and nucleocapsid gene sequences (*15*) by using Primer Express software version 1.5 or 2.0.0 (Applied Biosystems) with the following default settings: primer melting temperature (T_M_) set at 60°C; probe T_M_ set at 10°C greater than the primers at approximately 70°C; and no guanidine residues permitted at the 5′ probe termini. All primers and probes were synthesized by standard phosphoramidite chemistry techniques at the Biotechnology Core Facility at the Centers for Disease Control and Prevention (CDC). TaqMan probes were labeled at the 5′-end with the reporter molecule 6-carboxy-fluorescein and at the 3′-end with the quencher Blackhole Quencher 1 (Biosearch Technologies, Inc., Novato, CA). Optimal primer and probe concentrations were determined by crosstitration of serial twofold dilutions of each primer against a constant amount of purified SARS-CoV RNA. Primer and probe concentrations that gave the highest amplification efficiencies in this study were selected for further study (Table 1).

**Table 1:** Primers and probes used for real-time RT-PCR assays^a^

Assay ID	Primer/ probe	Sequence (5′>3′)	Genomic region	Location^b^
Primary diagnostic assay				
SARS1	F	CAT GTG TGG CGG CTC ACT ATA T	RNA polymerase	15370-15392
	R	GAC ACT ATT AGC ATA AGC AGT TGT AGC A		15422-15449
	P	TTA AAC CAG GTG GAA CAT CAT CCG GTG		15395-15420
SARS2	F	GGA GCC TTG AAT ACA CCC AAA G	Nucleocapsid	28531-28552
	R	GCA CGG TGG CAG CAT TG		28581-28597
	P	CCA CAT TGG CAC CCG CAA TCC		28559-28574
SARS3	F	CAA ACA TTG GCC GCA AAT T	Nucleocapsid	29016-29034
	R	CAA TGC GTG ACA TTC CAA AGA		29063-29083
	P	CAC AAT TTG CTC CAA GTG CCT CTG CA		29036-29061
To confirm positive results				
N3	F	GAA GTA CCA TCT GGG GCT GAG	Nucleocapsid	28432-28452
	R	CCG AAG AGC TAC CCG ACG		28383-28400
	P	CTC TTT CAT TTT GCC GTC ACC ACC AC		28406-28431
3′NTR	F	AGC TCT CCC TAG CAT TAT TCA CTG	3′ nontranslated region	29619-29642
	R	CAC CAC ATT TTC ATC GAG GC		29576-29595
	P	TAC CCT CGA TCG TAC TCC GCG T		29597-29618
M	F	TGT AGG CAC TGA TTC AGG TTT TG	Membrane protein	26951-26973
	R	CGG CGT GGT CTG TAT TTA ATT TA		27005-27027
	P	CTG CAT ACA ACC GCT ACC GTA TTG GAA		26974-27000

^a^RT-PCR, reverse transcription–polymerase chain reaction; F, forward primer; R, reverse primer; P, probe. ^b^Location based on the severe acute respiratory syndrome–associated coronavirus, Urbani strain (GenBank accession number AY278741).

### Real-Time RT-PCR Assay

The real-time RT-PCR assay was performed by using the Real-Time One-Step RT-PCR Master Mix (Applied Biosystems). Each 25-μL reaction mixture contained 12.5 μL of 2X Master Mix, 0.625 μL of the 40X MultiScribe and RNase Inhibitor mix, 0.25 μL of 10 μM probe, 0.25 μL each of 50 μM forward and reverse primers, 6.125 μL of nuclease-free water, and 5 μL of nucleic acid extract. Amplification was carried out in 96-well plates on an iCycler iQ Real-Time Detection System (Bio-Rad, Hercules, CA). Thermocycling conditions consisted of 30 min at 48°C for reverse transcription, 10 min at 95°C for activation of the AmpliTaq Gold DNA polymerase, and 45 cycles of 15 s at 95°C and 1 min at 60°C. Each run included one SARS-CoV genomic template control and at least two no-template controls for the extraction (to check for contamination during sample processing) and one no-template control for the PCR-amplification step. As a control for PCR inhibitors, and to monitor nucleic acid extraction efficiency, each sample was tested by real-time RT-PCR for the presence of the human ribonuclease (RNase) P gene (GenBank accesssion number NM_006413) by using the following primers and probe: forward primer 5′-AGATTTGGACCTGCGAGCG-3′; reverse primer 5′-GAGCGGCTGTCTCCACAAGT-3′; probe 5′-TTCTGACCTGAAG GCTCTGCGCG-3′. The assay reaction was performed identically to that described above except that primer concentrations used were 30 μM each. Fluorescence measurements were taken and the threshold cycle (C_T_) value for each sample was calculated by determining the point at which fluorescence exceeded a threshold limit set at the mean plus 10 standard deviations above the baseline. Clinical samples were considered positive if two or more of the SARS genomic targets showed positive results (C_T_ ≤45 cycles) and all positive and negative control reactions gave expected values.

Clinical specimens submitted to CDC for SARS-CoV testing that gave positive results were confirmed with a TaqMan real-time RT-PCR assay based on three different primer and probe sets (Table 1). This assay was performed independently in a separate laboratory using newly extracted nucleic acid from a second specimen aliquot. The confirmatory assay used the SuperScript One-Step RT-PCR (Invitrogen Corp., Carlsbad, CA) and the Mx4000 Multiplex Quantitative PCR system (Stratagene, La Jolla, CA).

### Synthesis of RNA Transcripts

Template for the nucleocapsid gene RNA was plasmid DNA (pCRII, Invitrogen Corp.) containing a full-length copy of the open reading frame for the SARS-CoV nucleocapsid gene oriented behind a T7 promoter. The plasmid was linearized by digestion with *Spe*I. The template for the polymerase RNA was a RT-PCR product generated by using the following primers: Cor-p-F2-T7, 5′-GTA ATA CGA CTC ACT ATA GGG CTA ACA TGC TTA GGA TAA TGG-3′ and Cor-p-R2, 5′-CCT ATT TCT ATA GAG ACA CTC-3′. Approximately 1 μg of RNA from Vero cells infected with SARS-CoV was used in RT-PCR reactions performed by using the SuperScript RT-PCR kit (Invitrogen Corp.) according to the manufacturer’s instructions; both templates were purified by phenol-chloroform extraction and ethanol precipitation before being used for in vitro transcription. RNA was synthesized in vitro by using the MegaScript kit (Ambion Inc., Austin, TX) according to the standard protocol. Synthetic RNA was treated with RNase-free DNase before being purified by phenol-chloroform extraction and ethanol precipitation. The concentration of RNA was determined by use of UV spectroscopy. Synthetic RNA was positive sense and 1,369 nt in length for N and 325 nt in length for polymerase.

## Results

### Real-Time RT-PCR Sensitivity and Reproducibility

Tenfold serial dilutions of the polymerase and nucleocapsid RNA transcripts were tested to assess the copy detection limits and dynamic range of our optimized real-time RT-PCR assays. The lower potential limit of detection was approximately 2 transcript copies per reaction for SARS2 and SARS3, and 7.5 copies per reaction for SARS1 (Figure). The confirmatory assays, which employ three different primer and probe sets (N3, 3′NTR, and M), showed potential limits of detection similar to the SARS2 and SARS3 assays. Strong linear correlations (r^2^ ≥0.99) were obtained between C_T_ values and transcript quantity over at least a 6-log range from approximately 10^2^ to 10^7^ copies per reaction for the three primer/probe sets. Linearity was markedly reduced for copy numbers exceeding 10^6^ (data not shown).

**Figure:**
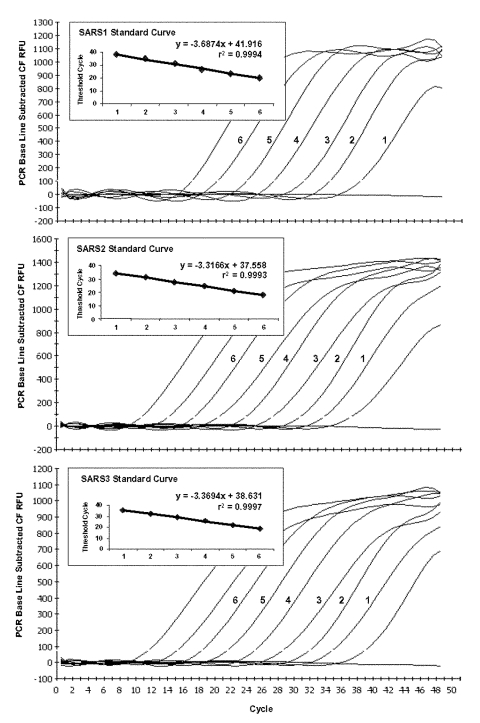
Typical amplification plot derived from serial 10-fold dilutions of severe acute respiratory syndrome–associated coronavirus RNA transcripts using TaqMan reverse transcription–polymerase chain reaction primer/probe sets SARS1, SARS2, and SARS3. A PCR Base Line Subtractive Curve Fit view of the data is shown with relative fluorescence units (RFU) plotted against cycle number. The default setting of 10 times the standard deviation of fluorescence in all wells over the baseline cycles was used to calculate the threshold cycle, or C_T_ value, for a positive reaction (horizontal line). Inserts show standard curve analysis of the RNA amplification plots with C_T_ values plotted against starting copy number. Plots derived from dilutions containing 2 x 10^6^ to 20 transcript copies for SARS2 and SARS3, and 7.5 x 10^6^ to 75 copies for SARS1.

Assay reproducibility was tested by using replicate 10-fold serial dilutions of the RNA transcripts and intra- and interassay variability evaluated for each dilution point in triplicate on three different days. At the lower copy detection limit for SARS2 and SARS3 (2 copies per reaction), assay reproducibility exceeded 90%. In contrast, the lower copy detection limit for SARS1 (7.5 copies per reaction) was positive in <50% of replicate reactions. One hundred percent reproducibility with SARS1 was achieved at the dilution that contained 75 transcript copies per reaction. Over the linear range of the assay, the coefficient of variation of the mean C_T_ values within and between runs was 0.46%–2.54% and 0.64%–2.39%, respectively (Table 2).

**Table 2:** Reproducibility of real-time RT-PCR assays^a^

	RNA transcript copy number^b^					
SARS1	7.5 x 10^1^	7.5 x 10^2^	7.5 x 10^3^	7.5 x 10^4^	7.5 x 10^5^	7.5 x 10^6^
CV within assay (%)^c^	2.53	0.96	0.49	0.69	1.66	0.7
CV between assays (%)^d^	2.39	1.09	0.82	0.64	2.1	0.79
SARS2	2.0 x 10^1^	2 x 10^2^	2 x 10^3^	2 x 10^4^	2 x 10^5^	2 x 10^6^
CV within assay (%)	1.27	0.57	0.46	0.72	0.84	0.67
CV between assays (%)	1.54	1.18	0.93	1.47	1.54	1.32
SARS3						
CV within assay (%)	0.8	0.55	0.65	0.5	0.27	1.25
CV between assays (%)	0.94	0.64	1.07	1.13	1.24	1.65

^a^RT-PCR, reverse transcription–polymerase chain reaction; CV, coefficient of variation. ^b^Ten-fold dilutions of the polymerase and nucleocapsid RNA transcripts; copies per reaction; dilution series thawed on 3 different days and assays performed in triplicate for each dilution. ^c^Determined from three replicates within each assay. ^d^Determined from three independent assays performed on different days.

To assess the efficiency of amplification of the RNA transcripts in the presence of exogenous nucleic acid and potential RT-PCR inhibitors, 10-fold serial dilutions of the RNA transcripts were prepared in water and pooled total nucleic acid extract from 20 SARS-CoV–negative human respiratory specimens (nasopharyngeal aspirates, bronchial washes, sputum, naso- and oropharyngeal swabs, and lung tissue). Exogenous nucleic acid had no discernible effect on amplification efficiency of the SARS1 and SARS3 primer/probe sets, as demonstrated by the similarity in linear regression slopes and endpoint detection limits in the presence and absence of specimen extract (Table 3). In contrast, the standard curve for SARS2 had a more efficient slope (–3.21) in water than in the presence of spiked extract (–3.48) and with greater variation in the C_T_ values at 20 target copies or lower, suggesting that the amplification reaction was less efficient in the presence of the specimen extract. This observation was confirmed on two additional repetitions of the same experiment.

**Table 3:** Efficiency of real-time PCR assays^a^

	Mean C_T_^b^ values at estimated RNA transcript copy number									
SARS1	7.5 x 10^0^	7.5 x 10^1^	7.5 x 10^2^	7.5 x 10^3^	7.5 x 10^4^	7.5 x 10^5^	7.5 x 10^6^	Slope^c^	Efficiency (%)^d^	
RNA transcript alone	Neg	38.65±1.48	34.25±0.57	31.1±0.14	27.5	24.2	20.55±0.07	–3.55	91.1	
RNA transcript + extract^e^	Neg	38.05±0.92	34.85±0.21	31.55±0.07	27.75±0.07	24.4	20.6	–3.49	93.3	
SARS2	2 x 10^0^	2 x 10^1^	2 x 10^2^	2 x 10^3^	2 x 10^4^	2 x 10^5^	2 x 10^6^			
RNA transcript alone	35.4±0.57	32.1±0.14	29.45±0.64	26.15±0.07	22.9±0.14	19.4	16.35±0.07	–3.21	104.9	
RNA transcript + extract	Neg	34.55±1.91	29.2±0.28	26.2	23.1	19.6±0.14	16.6	–3.48	93.9	
SARS3										
RNA transcript alone	39.3	36.2±0.42	32.8	29.1±0.14	25.9	22.15±0.07	19.2	–3.39	97.1	
RNA transcript + extract	40.3	36.2±0.28	33.4±0.28	29.9±0.21	26.05±0.07	22.55±0.21	19.65±0.21	–3.42	96.1	

^a^PCR, polymerase chain reaction; CT, threshold cycle number. ^b^Values shown are mean of triplicate samples ± standard deviations. ^c^Slope determined from the formula: Y = Y intercept – slope log_10._ Slopes calculated for SARS1 (7.5 x 10^6^ to 7.5 x 10^1^); SARS2 (2 x 10^6^ to 2 x 10^1^); SARS3 (2 x 10^6^ to 2 x 10^0^). ^d^Efficiency = [10^(–1/slope)^] – 1. ^e^Reactions performed in presence of pooled total nucleic acid extract from 20 human respiratory specimens.

The real-time RT-PCR assay was compared with a previously described conventional RT-PCR for SARS-CoV by using fluorescent dye-labeled primers and GeneScan amplicon analysis (*5*). Tenfold serial dilutions of a pretitrated SARS-CoV stock adjusted to 1 x 10^7^ PFUs/mL were prepared in triplicate and tested by all assays (Table 4). The real-time RT-PCR assays were positive with 100% frequency at a 10^–8^ dilution. Accordingly, the lowest virus quantity detected was 0.01 PFU/100 μL of specimen extract. The conventional RT-PCR assay was at least 10-fold less sensitive in repeat comparisons.

**Table 4:** Comparison of real-time RT-PCR assays with culture and conventional RT-PCR^a^

SARS-CoV dilution^b^	Conventional RT-PCR	Real-time RT-PCR		
		SARS1	SARS2	SARS3
10^–4^	3/3^c^	3/3	3/3	3/3
10^–5^	3/3	3/3	3/3	3/3
10^–6^	3/3	3/3	3/3	3/3
10^–7^	3/3	3/3	3/3	3/3
10^–8^	0/3	3/3	3/3	3/3
10^–9^	0/3	0/3	1/3	0/3
10^–10^	0/3	0/3	0/3	0/3

^a^RT-PCR, reverse transcription–polymerase chain reaction; SARS-CoV, severe acute respiratory syndrome–associated coronavirus. ^b^Serial 10-fold dilution of SARS-CoV stock culture containing 1 x 10^7^ PFUs/mL. ^c^Number of positive results divided by the number of replicates tested.

### Specificity

We compared our primer and probe sets with sequences for 14 SARS-CoV field isolates that became available during the course of this study (*16*) and found no nucleotide mismatches. In contrast, alignments with other published human and animal coronaviruses (GenBank accession no.: human coronaviruses X69721 and AF124989; bovine coronaviruses NC003045 and AF124985; murine hepatitis viruses NC001846 and M55148; sialodacryoadenitis virus AF124990; canine coronavirus AF124986; feline infectious peritonitis virus AF124987; porcine hemagglutinating encephalomyelitis virus AF124988, Z34093, and AF124992; turkey coronavirus AF124991; and avian infectious bronchitis virus NC_001451) showed little sequence identity with our primer and probe sets. To further assess the potential for crossreactions with other members of the *Coronaviridae* family, the RT-PCR assays were tested against nucleic acid extracts of human respiratory coronaviruses OC43 (VR-759) and 229E (VR-740), feline infectious peritonitis virus (VR-3004), mouse hepatitis virus (VR-1426), bovine coronavirus (VR-874), porcine transmissible gastroenteritis virus (VR-743), and avian infectious bronchitis virus (VR-841), obtained from the American Type Culture Collection (Manassas, VA), and human enteric coronavirus (VR-1475). In addition, nucleic acid extracts of field isolates of influenza A and B; parainfluenza 1, 2, and 3; rhinovirus; adenovirus; human metapnuemovirus; and respiratory syncytial virus, as well as human and nonhuman primate cell lines were tested. No positive reactions were obtained with any of the primer and probe sets.

### Evaluation with Clinical Specimens

The real-time RT-PCR assay was used to test 14 clinical specimens (including throat swab [2 specimens], sputum [1 specimen], throat wash [5 specimens], and lung autopsy tissues [6 specimens]) from 10 patients with laboratory confirmed SARS-CoV infection (Table 5). Assay results were positive with all specimens for all three primer/probe sets. In addition, 326 respiratory specimens collected during the course of the outbreak from 236 suspected U.S. SARS patients who were serologically negative for SARS-CoV infection were also negative by the real-time RT-PCR.

**Table 5:** Results of real-time RT-PCR assay with specimens from patients with laboratory-confirmed SARS-CoV infection^a^

Case ID	Location	Specimen ID	Specimen	Serology	Vero E6 culture	Conventional RT-PCR^b^	Real-time RT-PCR C_T_^c^ values			
							SARS1	SARS2	SARS3	RNase P
05078	Toronto	2003756523	Lung, RM	N/A	–	+	24.2	21.6	23	23.9
		2003756525	Lung, RU		–	+	24.9	21.5	23	23.7
05077	Thailand	2003756502	Throat swab	+	+	+	37.5	36.2	39.8	29.3
05000	Hong Kong	2003757035	Lung, RU	+	–	+	26.7	22.6	24.1	24.7
		2003757036	Lung, LU		–	+	27.2	24.9	26.5	26
		2003757037	Lung, RM		–	+	34.9	37.5	31.9	27.4
		2003757038	Lung, LL		–	+	29.6	27	28.6	24.5
00220	Utah, USA	2003757508	Sputum	+	+	+	24.7	23	24.8	30.6
05001	Vietnam	2003757190	Throat wash	+	+	+	23.7	22.4	24.1	30.1
05008	Vietnam	2003757229	Throat wash	+	–	+	35.5	35.5	36.7	30
05010	Vietnam	2003757239	Throat wash	+	–	+	31.1	29.3	31.5	34.2
05013	Vietnam	2003757251	Throat wash	+	–	+	29.5	28.4	30.3	28.8
05017	Vietnam	2003757268	Throat wash	+	+	+	26	24.7	26.4	27.9
05316	Vietnam	2003759760	Throat swab	N/A	+	N/A	25	25.3	28.2	28

^a^RT-PCR, reverse transcription–polymerase chain reaction; SARS-CoV, severe acute respiratory syndrome–associated coronavirus; CT, threshold cycle number; RM, right middle; RU, right upper; LU, left upper; LL, left lower; N/A, not applicable. ^b^Ref. 5. ^c^Values shown mean of duplicate values.

## Discussion

In response to the SARS outbreak, we developed a real-time RT-PCR assay based on multiple primer and probe sets designed to different genomic targets to facilitate sensitive and specific detection of SARS-CoV in clinical specimens. A potential detection limit of <10 transcript copies was achieved with greater relative sensitivity than cell culture isolation or conventional RT-PCR. The potential for quantitation over a wide dynamic range (at least 6 logs) was demonstrated with low intra- and interassay variability and limited inhibition from exogenous nucleic acid extract from respiratory secretions. The increased sensitivity of the real-time RT-PCR assay over cell culture and conventional RT-PCR methods may aid detection of the virus at earlier stages of infection, when the virus is present at low titer in respiratory secretions (*8*). In addition, by eliminating the need for postamplification product processing, the real-time RT-PCR format permitted shortened turnaround time for reporting results, which proved critical during the SARS outbreak.

Although real-time RT-PCR offers clear advantages over more conventional RT-PCR formats, assay results must still be interpreted with caution. For example, the effectiveness of RT-PCR for detection of SARS-CoV in clinical specimens has been shown to be greatly influenced by the quantity, type, and timing of specimen collection (*8*,*17*). False-negative results due to poor quality nucleic acid or presence of RT-PCR inhibitors can also be a concern. We addressed this by simultaneously testing for the human RNase P gene, which should be present in all adequately collected samples. False-negative results could also potentially arise from mutations occurring in the primer and probe target regions in the SARS-CoV genome. We addressed this by including multiple genetic targets in our assay and by carefully comparing our primer and probe sequences against published sequences of SARS-CoV as they became available. To avoid false-positive results, meticulous care was taken to prevent introduction of contaminating viral RNA or previously amplified DNA during preparation of the nucleic acid extracts and amplification reactions. In addition, all RT-PCR–positive specimens were retested from a second, unopened sample aliquot and confirmed in a second laboratory by using a real-time assay based on different genetic targets.

In conclusion, our real-time RT-PCR assay permitted rapid, sensitive, and specific detection of SARS-CoV in clinical specimens and provided needed diagnostic support during the recent SARS outbreak. Widely deploying this assay through the LRN will enhance our ability to provide a rapid response in the event of the possible return of SARS.
